# Traditional uses of medicinal animals in the semi-arid region of northeastern Brazil

**DOI:** 10.1186/1746-4269-8-41

**Published:** 2012-10-10

**Authors:** Rômulo Romeu Nóbrega Alves, Rita Oliveira de Sousa Neta, Dilma Maria de Brito Melo Trovão, Jose Etham de Lucena Barbosa, Adrianne Teixeira Barros, Thelma Lucia Pereira Dias

**Affiliations:** 1 Departamento de Biologia, Universidade Estadual da Paraíba, Av. das Baraúnas, 351/Campus Universitário, Bodocongó, 58109-753, Campina Grande, PB, Brazil

## Abstract

The present work presents an inventory of the traditional medicinal uses of animals in the municipality of Bom Sucesso in Paraíba State (PB) in the semiarid northeastern region of Brazil. Information was obtained through the use of semi-structured interviews with 50 people who use zootherapeutic products. A total of 25 animal species used for medicinal purposes were identified (18 vertebrates and seven invertebrates) distributed among five taxonomic categories; the groups with the largest numbers of citations were: mammals (8 citations), insects (7), and reptiles (5). The most cited animal species were: *Tubinambis merianae* “teju” lizards (44 citations); *Apis mellifera* Italian honeybees (318 citations); *Gallus gallus* chickens (31 citations); *Ovis aries* sheep (31 citations); *Crotalus durissus* rattlesnakes (14 citations); *Boa constrictor* (12 citations); and *Bos taurus* cattle (12 citations). A significant number of illnesses and conditions treated with animal-based medicines were cited, and the category with the greatest number of citations was “problems affecting the respiratory system”. Our results suggest that the use of zootherapeutics in the region is persistent, and that knowledge about these curative practices is an integral part of the regional culture. As such, studies concerning the uses of zootherapeutics are important windows to understanding human/environmental/cultural interactions and a pathway to conciliating regional cultures with efforts to conserve the native fauna.

## Background

Humans have always used nature as a source of basic resources for survival, including treatments for infirmities, and human medical practices include the use of plants, animals, and minerals in the production of remedies [1]. Researchers have generally emphasized studies of medicinal plants, relegating medicinal animals a lower priority. Recently, however, investigations of this theme have intensified, reinforcing the idea that the use of medicinal animal resources has long been present in all societies and continues even up to the present day [2-15].

Brazil’s high biological and sociocultural diversity [16,17] translates into a wealth of traditional knowledge and practices, including the use of animals for medicinal purposes. It is estimated that Brazil hosts between 15% and 20% of the world’s biological diversity, and the greatest number of endemic species. Brazil has more than 200 indigenous tribes and a large number of traditional communities that all possess considerable knowledge about the local fauna and flora and exhibit an array of natural resource use strategies and each ethnic culture possesses a broad knowledge regarding the medicinal properties of wildlife species [18,19]. The extensive medicinal use of animal parts and products has been documented both in rural and urban areas [3,4,6,17-31], and is sustained by a thriving trade in medicinal animals conducted by herbalists in markets throughout Brazil [3,4,17,32].

Studies of the utilization of medicinal animals by human populations living in northeastern Brazil are still relatively scarce, even though the region retains significant cultural and biological diversity that is reflected in a rich popular knowledge about biological medicinal resources [24]. Historically, ethnobiological studies have been largely directed towards ethnobotanical themes, although ethnozoological studies have intensified in recent years [33]. In the specific case of the semiarid region of Paraíba State, studies undertaken in a number of localities [11,30,34-36], have highlighted the cultural importance of using zootherapeutic items for treating human as well as animal illnesses and reflect the strong relationship these local populations have with the natural resources available around them [37,38]. In an effort to contribute to our knowledge of zootherapeutic practices and their implications in the semiarid region of northeastern Brazil, the present work documented the animals used as zootherapeutics in the municipality of Bom Sucesso in the semiarid region of Paraíba State (PB).

## Methods

**Figure 1:**
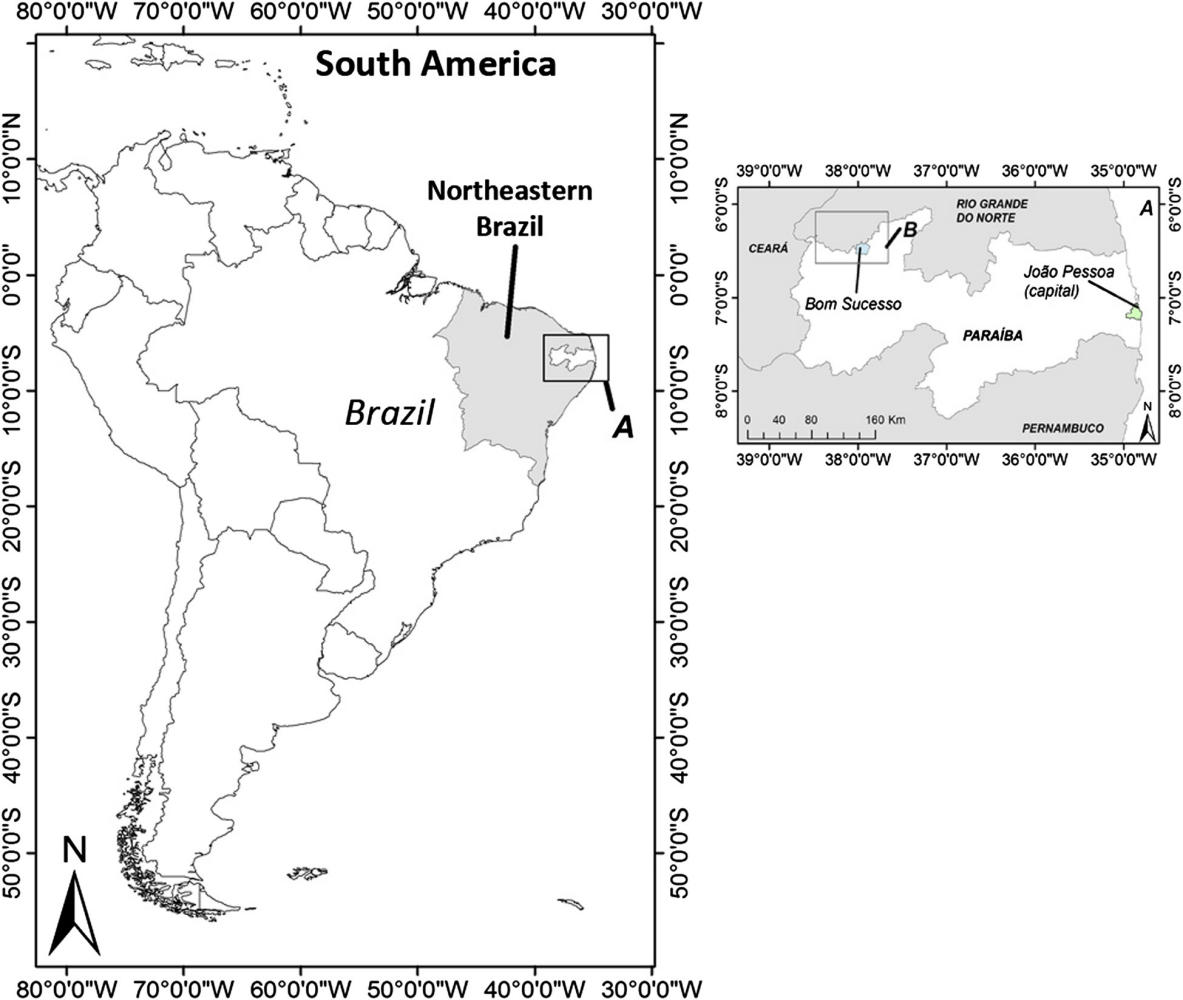
Location of Bom Sucesso municipality, Paraíba State, Northeastern Brazilian region.

The present research was carried out in the municipality of Bom Sucesso (06° 26' 42'' S; 37° 55' 46'' W), situated in western Paraíba State in northeastern Brazil (Figure 1). This municipality was created by State Law N° 3049 of June 17, 1963, and comprises an area of 198 km^2^, with a population of 5,280 inhabitants, of which 1,558 live in urban areas and 3,722 in the rural zone.

The municipality of Bom Sucesso is located within the semiarid region known as the Drought Polygon of Brazil. The regional climate is hot and humid, with rainfall in the autumn and winter (classified as AW' by the Koppen system). Paraíba State is divided into bioclimatic regions, and the municipality of Bom Sucesso has a hot and attenuated dry climate, with 7 to 8 months of drought, average yearly temperatures of 27 to 28°C, and with average annual rainfall of 1,000 mm (type 4ath-Tropical). The regional vegetation is known as *Caatinga* (deciduous thorny scrub) and the local topography varies from 270 to 600 m above sea level. The municipal health infrastructure includes a hospital with 16 beds and three ambulatory units. There are 16 grammar schools and one high school.

### Procedures

Field research was conducted from December 2007 to February 2008. During the first contacts with the local population, we attempted to identify local people with a specialized knowledge of medicinal animal use, following Alves and Rosa [20]. A specialist is defined as “a person recognized by the community as having deep knowledge about the use of animals in manufacturing remedies and in promoting cures”. Information on the use of animal in traditional medicines was collected through interviews with 50 persons (11 men and 39 women), mainly from the elderly people, who still retain the major portion of traditional knowledge in their respective communities. Additional interviewees were chosen by using the snowball technique, based on information initially provided by the specialists. Interviews were conducted on a one-to-one basis. Prior informed consent was obtained for all interviews conducted.

Data were gathered through interview-questionnaires, with some questions left open-ended [39]. Questionnaires encompassed the following aspects: local name of the animal used as remedy; parts used as medicine; conditions treated with the remedy; preparation and usage; restrictions of use; spiritual aspects linked to the use; use of live or dead animals; how animals were obtained; storage conditions; collection sites; efficacy of the remedies; traditional uses of the remedies in the community; how knowledge was acquired by the interviewees; reliance on animal-based remedies; why the interviewee used animal-based remedies.

The ethical approval for the study was obtained from the Ethics committee of Paraiba University State. To respect intellectual property rights, we adopted the following protocol in the field: before the survey, we introduced ourselves, explained the nature and objectives of our research and asked the respondents for permission to record the information.

Species’ vernacular names were recorded as quoted by interviewees. Zoological material was identified with the aid of specialists, through (1) examination of voucher specimens donated by the interviewees; (2) photographs of the animals or their parts, taken during interviews; (3) vernacular names, with the aid of taxonomists familiar with the study areas’ fauna. Voucher specimens and/or photographs were deposited at the Department of Systematics and Ecology, Federal University of Paraíba.

### Data analysis

#### Use value

For each species was calculated the use value (adapted from the proposal of Phillips et al. [40], a quantitative method that demonstrates the relative importance of species known locally. These value was calculated using the following formula: UV = *Σ*U/n, where: UV = use value of a species; U = number of citations per species; n = number of informants.

Application of the use-value of each species is based objectively on the importance attributed by the informants and does not depend on the opinion of the researcher.

## Results and discussion

**Figure 2:**
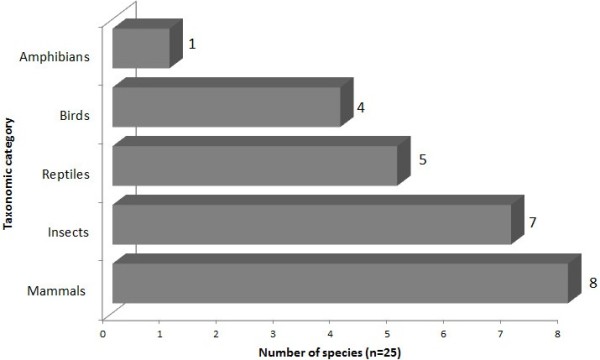
Animal species used as remedies by taxa in Bom Sucesso, Paraíba State, Northeastern Brazil.

A total of 25 medicinal animals were cited by the interviewees (21 vertebrates and seven invertebrates). These species inventoried include five taxonomic groups: mammals (8 species), insects (7 species), birds (4 species), reptiles (5 species), and amphibians (1 species) belonging to 19 families (Table 1 and Figure 2).

**Table 1:** Animals species used in popular medicine in the municipality of Bom Sucesso, Paraíba State, Brazil

**Family/species/local name**	**Number of citation**	**Use Value**	**Part used**	**Disease (or illness)**
INSECTS				
Apidae				
*Apis mellifera* (Linnaeus, 1758)–Africanised honey bee, “abelha italiana”	38	0.56	Honey (7,8)	Sore throat, flu, shortness of breath, cough, tuberculosis
*Partamona cupira* (Smith)–a stingless Bee, “Abelha cupira”	2	0.04	Honey (7,8)	Earache, menstrual cramps
*Melipona subnitida* (Ducke, 1910)–jandaíra	12	0.16	Honey (7,8)	Sore throat, flu, earache, hoarseness
*Tetragonisca angustula* (Latreille, 1811)–abelha mosquito	6	0.10	Honey(7,8)	Earache, sore throat
*Cephalotrigona capitata* (Smith 1854)–Abelha mumbuca	2	0.02	Honey (7,8)	Sore throat, nervous problems
Termitidae				
*Nasutitermes macrocephalus* (Silvestri, 1903)–Termite, Cupim	2	0.02	Whole anima (7)	Bronchitis, whooping cough
Unidentified family				
Unidentified species-beetle	1	0.02	Beetle’s Nest	Mumps
AMPHIBIANS				
Bufonidae				
*Rhinella jimi* (Stevaux, 2002), Toad	1	0.02	Abdomen (10)	Erysipelas
REPTILES				
Chelidae				
*Phrynops geoffroanus* (Schweigger, 1812)–Geoffroy’s side-necked turtle, “cágado”	5	0.06	Fat (3)	Swelling, sore throat, flu, stuffy nose
Teiidae				
*Tupinambis merianae* (Duméril & Bibron, 1839)–Teju lizard, “tegu”, “tejuaçú”	44	0.88	Fat (3), meat (5)	Sore throat, thrombosis
Crotalidae				
*Crotalus durissus* Linnaeus, 1758–South American rattlesnake, “Cascavel”	14	0.22	Fat (3)	Rheumatism, skin spots, eye problems, plantar fasciitis, Swelling, pain in general, hoarseness
Boidae				
*Boa constrictor* (Linnaeus, 1758)–Boa, Cobra de veado	12	0.22	Fat (3)	Rheumatism, swelling, herniated intervertebral disk, bone fractures
Tropiduridae				
*Tropidurus hispidus* (Spix, 1825)–Lizard, Lagartixa	1	0.02	Whole animal (9)	Sore throat
BIRDS				
Phasianidae				
*Gallus gallus domesticus* (Linnaeus, 1758)–chicken, Galinha	31	0.38	Gizzard (5), fat (3 or 8), eggshells (2), egg yolk (6), meat (5)	Indigestion, sinusitis, shortness of breath, bronchitis, nervous problems, rheumatism, stuffy nose, weak bones, flu, weakness, sore throat, furuncle
Meleagrididae				
*Numida meleagris* Linnaeus, 1758–Helmeted Guineafowl, “Guiné”	2	0.04	Meat (5)	whooping cough
Anatidae				
*Anas platyrhynchos* Linnaeus, 1758–Duck, Pato	1	0.02	Eggs (5)	Weakness
Corvidae				
*Cyanocorax cyanopogon* (Wied-Neuwied, 1821)–White-naped Jay	1	0.02	Gizzard (5)	Asthma
MAMMALS				
Trichechidae				
*Trichechus manatus* (Linnaeus, 1758)–Manatee, Peixe-boi	3	0.02	Fat (3)	Headache, rheumatism, corporal lesions
Canidae				
*Canis lupus* (Linnaeus, 1758)–Domestic dog, Cachorro	1	0.02	Faeces (1)	Measles
Dasypodidae				
*Dasypus novemcinctus* (Linnaeus, 1758)–Nine-banded armadillo, Tatu-verdadeiro	2	0.04	Meat (5), tail (4)	Back ache, snake bite
Mustelidae				
*Conepatus semistriatus* (Boddaert, 1785)–Striped hog-nosed skunk, “Ticaca”	1	0.02	Meat (5)	Rheumatism
Caviidae				
*Kerodon rupestris* (Wied-Neuwied, 1820)–Rock cavy, “Mocó”	3	0.04	Meat (5)	Disorders after parturition (to accelerate recovery after parturition), weakness, thrombosis
Bovidae				
*Bos taurus* Linnaeus, 1758–Domestic cattle, “Boi”	12	0.20	Liver (5), horn (1,7), marrow (7), milk (8), urine (8), butter (8), hoof proteins (7)	Anaemia, the evil eye, nervous problems, whooping cough, weakness, eye problems, sore throat, baldness, tuberculosis
*Ovis aries* Linnaeus, 1758–Sheep, “Carneiro”	31	0.36	Fat (4), suet (4)	Rheumatism, cracks in the sole of the feet inflammations, swelling, nervous problems, furuncle, one fractures, suck a splinter out of skin or flesh, *mycosis*
Hydrochaeridae				
*Hydrochoerus hydrochaeris* (Linnaeus, 1766), capybara, capivara	1	0.02	Fa (3)	Osteoporosis

Legend: 1, Tea; 2, powder, to be ingested with food; 3, ointment to be rubbed on the affected area; 4, ear drops; 5, ingestion of the cooked part; 6, ingestion of the crude part; 7, beverage (‘garrafada’), obtained by mixing medicinal plants and animals; 8, taken as a drink; 9, concoction, taken as a drink; 10, Rubbed on the affected area.

**Figure 3:**
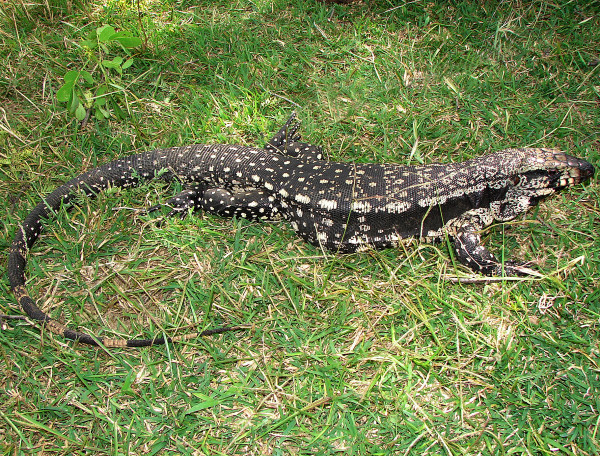
***Tubinambis merianae*, the most important medicinal animal in the municipality of Bom Sucesso, Paraíba State, Brazil.**

The most cited animal species were: *Tubinambis merianae* (Duméril & Bibron, 1839 (Figure 3)) “teju” lizards (44 citations); *Apis mellifera* (Linnaeus, 1758) Italian honeybees (28 citations); *Gallus gallus* (Linnaeus, 1758) chickens (19 citations); *Ovis aries* (Linnaeus, 1758) sheep (17 citations); *Crotalus durissus* (Linnaeus, 1758) rattlesnakes (11 citations); *Boa constrictor* (11 citations); and *Bos taurus* (Linnaeus, 1758) cattle (10 citations).

As has been seen in other studies of zootherapeutics undertaken in Brazil [41] many animals had multiple medicinal uses, such as: cattle (*Bos taurus)*, whose body parts (liver, urine, butter, marrow, hoof proteins) are used to treat illnesses such as sore throats, anemia, and severe coughing (whooping cough), and; chickens (*Gallus domesticus*), whose body parts (fat, gizzard, eggs [shells and yolks], and meat) are used to treat sinus problems, shortness of breath, indigestion, bronchitis, stuffy nose, rheumatism, weak bones, colds, general weakness, sore throats, hoarseness, and boils. Among the species with multiple therapeutic indications are *Apis mellifera*, *Ovis aries*, and *Crotalus durissus.*

The accessibility and availability of local faunal resources influence the choices of the zootherapeutics utilized, so that the majority of the medicinal animals used occur within the research area. The commerce of zootherapeutic items in public markets is very common in many Brazilian towns and cities [4,5,7,32,42,43], which results in additional pressure on useful species, although the results of our interviews indicated that relatively few people in the region bought their zootherapeutic items, being able to obtain these substances from animals captured and/or kept near their homes.

The medicinal animals cited in this survey were used to treat 43 different illnesses and maladies (Table 1). The treatment categories with the largest number of citations were: problems affecting the respiratory apparatus and illnesses of the osteomuscular system – which corroborates the results of other research projects that have examined the use of zootherapeutics. Alves and Rosa [20] demonstrated that problems affecting the respiratory system and the osteomuscular system and connective tissues always appeared as the most cited in investigations of home-remedy uses in northern and northeastern Brazil. Costa-Neto [26] likewise found that animal-based medicines are frequently used to treat respiratory diseases in Bahia State, in northeastern Brazil. Similar results were reported by Silva et al. [44] in a survey of public markets in the city of Recife in Pernambuco State (also in the northeastern region of that country).

Home remedies are obtained from whole animals, or their body parts, or products extracted from them, such as fat, honey, milk, butter, wax, urine, feces, meat, skin, bones, liver, tails, gizzards, and eggs. Among these products, animal fat was the most cited in this and in other studies [2,5-7,14,18,20,25,32,35]. Most of these materials have been reported in other surveys of human utilization of zootherapeutic resources in Brazil [14,24,45], indicating that these practices are widely disseminated throughout the country.

The zootherapeutic resources identified in the present study demonstrated Use-values (VU) that varied from 0.02 to 0.88. Most of the species surveyed had low VU values, with 67% percent of them being below 0.10 (Table 1). These data indicates considerable variation in species uses, with some species being frequently utilized and therefore more important in traditional local medicine. The species having the greatest use-values were *Tupinambis merianae* (VU = 0.88), *Apis mellifera* (VU = 0.56), and *Gallus domesticus* (VU = 0.38), and all three are known to be widely used in traditional medicine throughout Brazil [14,24,45].

One important aspect of the use of medicinal animals is related to public health [15,20]. In the present study, which involved many home visits, we witnessed inappropriate storage of many products and only minimal considerations of hygiene. These lax storage practices did not seem to concern most of the people using these materials, and they demonstrated a lack awareness of the possible grave health consequences of inadequate storage of zootherapeutics (or any other product used for medicinal purposes). In their research on zootherapy in northern and northeastern Brazil, Alves and Rosa [32], likewise observed a general lack of concern for the storage conditions of zootherapeutic products and the risks of bacterial contamination, and Alves and Rosa [15] recommended the implementation of sanitary measures to govern the use and storage of these folk medicines in light of possible negative effects on public health.

Our results indicated that the use of animals for medicinal purposes in the community studied was quite accentuated, and that these people retain considerable knowledge about the local fauna used in health care treatments. It was also seen that zootherapeutics are most frequently used to treat the common illnesses in the population, such as problems affecting the respiratory apparatus (including throat inflammations, coughing, colds, and asthma). Popular knowledge about these curative practices is an integral part of the regional culture and demonstrates the necessity of carefully studying zootherapeutic practices to better understand human/environmental/cultural interactions – especially in light of the probable existence of valuable pharmacologically active substances in these remedies and the opportunity to conciliate the regional culture with animal conservation efforts.

The results obtained in the present research, together with data provided by other workers, confirm that the medicinal uses of animals represent ethnozoological connections of relevant cultural value in the semiarid region of northeastern Brazil. Additional studies will be necessary to amplify our knowledge of the regional medicinal fauna from a historical perspective and examine the interwoven cultural, pharmacological, and ecological interdependencies of zootherapeutic practices in traditional cultures.

## Competing interests

The authors declare that they have no competing interests.

## Authors’ contributions

RRNA, ROSN, DMBMT, JEL, ATB and TLPD-Writing of the manuscript, literature survey and interpretation; RRNA and ROSN-Ethnozoological data, and analysis of taxonomic aspects. All authors read and approved the final manuscript.
